# The underrated prevalence of depression in Japanese patients with rheumatoid arthritis - evidence from a Nationwide survey in Japan

**DOI:** 10.1186/s41927-017-0003-6

**Published:** 2017-11-28

**Authors:** Rosarin Sruamsiri, Yuko Kaneko, Jörg Mahlich

**Affiliations:** 1 Health Economics, Janssen Pharmaceutical KK, 5-2, Nishi-kanda 3-chome Chiyoda-ku, Tokyo, 101-0065 Japan; 2 Center of Pharmaceutical Outcomes Research, Naresuan University, Phitsanulok, Thailand; 3 Division of Rheumatology, Department of Internal Medicine, Keio University School of Medicine, Tokyo, Japan; 4 Düsseldorf Institute for Competition Economics (DICE), University of Düsseldorf, Düsseldorf, Germany

**Keywords:** Depression, Rheumatoid arthritis, Prevalence, PHQ-9

## Abstract

**Background:**

To determine the prevalence of depression among Japanese people with rheumatoid arthritis (RA) and explore the relationships between depression and an array of variables.

**Methods:**

Nation-wide, cross-sectional online survey (*n* = 500) of people with RA including the Patient Health Questionnaire (PHQ-9) to measure the presence and severity of depressive symptoms were performed.

**Results:**

While only 5% of the population studied had been officially diagnosed with depression, 35% had PHQ-9 scores indicating depression was present. People with RA are more likely to experience depression if they are younger, have greater functional impairment, or whose treatment regimen includes pain medications not biologic agents.

**Conclusions:**

It is a potential risk of under-diagnosis and under-reporting of depression in Japanese people with RA. People with RA are more likely to experience depression if they are younger, have greater functional impairment, or whose treatment regimen includes pain medications without biologic drugs.

**Electronic supplementary material:**

The online version of this article (10.1186/s41927-017-0003-6) contains supplementary material, which is available to authorized users.

## Background

Significant evidence in the scholarly literature suggests that depression is a common comorbidity among patients with rheumatoid arthritis (RA) [1–3]. The prevalence of depression varies significantly between different studies, but by some estimates occurs in as many as 42% of RA patients [4]. A meta-analysis of 72 studies involving 13,189 people with RA revealed that from 14.8 to 38.8% of people with RA receive a diagnosis of a major depressive disorder [5].

Depression in RA is a risk factor not only for suicidal ideation [6] but for cardiovascular disease [7], myocardial infarction [8], and mortality [9]. Patients with RA are at greater risk of experiencing anxiety, depression, and low self-esteem, with higher levels of associated mortality and suicide [10]. It was suggested that nearly 11% of people with RA experience suicidal thoughts, a statistic that rises to an alarming 30% in patients with a co-morbidity of depression [11]. Previous study found that people with comorbid RA and depression may use more health services and are less likely to adhere to their medication regimens [12, 13]. A comorbidity of RA and depression is also associated with higher unemployment, work productivity losses, and increased healthcare costs to both individual patient and society [13, 14].

More often than not comorbid depression with RA is undiagnosed and therefore untreaeted [15] often because rheumatologists and their patients seldom communicate about depression [13, 16]. In the USA and Europe, the prevalence of depression was high in people with RA and the prevalence was found to be greater in younger patients [5]. Similar findings were found in only one Japanese study that identified factors associated with depression in female people with RA and found that among sociodemographic factors, “a decrease in the frequency of going out socially after having RA” and “a higher education” were significantly associated with depression [17]. However, evidence of depression in people with RA is limited in Asia studies and also Japan.

The present study was undertaken to determine the prevalence of depression in Japanese People with RA and explore the relationships between RA patient, depression and an array of demographic variables.

## Methods

### Patient population and data collection

**Fig. 1:**
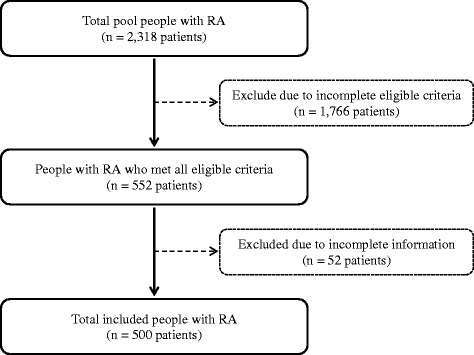
Patient flow

A nationwide cross-sectional online survey was carried out in Japan during the months of July and August in 2016. The survey was national in its scope, drawing from a pool of more than 2000 people with RA. Figure 1 showed patient flow of this study. The study included 500 people with RA who had been diagnosed with RA for at least 1 year, whose current treatment scheme included at least one RA medication and completed all questionnaires.

The survey instrument contained questions about a variety of patient demographic characteristics such as age, gender, marital status, education, employment, and income. It also included questions covering clinical characteristics such as length of time since diagnosis, functional impairment as assessed using the Japanese version of the Stanford Health Assessment Questionnaire (J-HAQ score) [18], and the participant’s current medical treatment scheme including whether biologic agents and conventional synthetic disease modifying anti-rheumatic drugs (conventional synthetic disease-modifying antirheumatic drugs; csDMARDs) are being used.

The Patient Health Questionnaire with 9 items (PHQ-9) was used to measure the presence and severity of depressive symptoms. The study used a Japanese language version of the PHQ-9 shown to be a valid screening tool to detect potential depression patients in Japanese hospitals [19]. In addition, the similar findings have been shown when using the PHQ-9 web-base and paper based survey [20]. The cut-off scores were defined as follows: no depression (0–4), mild (5–9), and moderate-to-severe (≥10) [21].

### Statistical analysis

Descriptive statistics were used to quantify baseline characteristics and depression condition while one-way analysis of variance was used to determine differences among relevant patient groupings. Ordered logistic regression was used to identify the determinants of depression conditions among survey respondents. We also reported univariate correlation coefficients between depression conditions and all other variables to assess associations of depression condition with each factor. In the multivariate model, all factors with *p* values in the univariate analyses of *p* < 0.2 were included (stepwise regression). All analyses were calculated using STATA version 14.0 (College Station, TX, USA). A *P* value of <0.05 was considered statistically significant.

## Results

### Patient demographics

Data from a total of 500 patients with RA were included in the analysis. The mean age was 54.3 years old and 67% of the patients were female (Table 1).

**Table 1:** Depression in RA patients analyzed by demographic characteristics

Characteristics	Overall, N (%)	No depression, N (%)	Mild depression, N (%)	Moderate to severe depression, N (%)	*P*-value
Patients	500	324 (65)	118 (24)	58 (11)	
Age (mean ± SD)	54.28 ± 10.02	56.04 ± 9.91	51.57 ± 10.02	49.93 ± 9.59	<0.001
≤ 50 years	178 (36)	94 (29)	53 (45)	31 (54)	
51–60 years	196 (39)	130 (40)	45 (38)	21 (36)	
> 60 years	126 (25)	100 (31)	20 (17)	6 (10)	
Gender					0.036
Male	163 (33)	118 (37)	28 (24)	17 (29)	
Female	337 (67)	206 (64)	90 (76)	41 (71)	
Marital status					0.004
Single	97 (19)	49 (15)	34 (29)	14 (24)	
Married	403 (81)	275 (85)	84 (71)	44 (76)	
Highest Education					0.109
High school or less	180 (36)	107 (33)	47 (40)	26 (44)	
College	120 (24)	74 (23)	30 (25)	16 (28)	
Bachelor’s degree or higher	200 (40)	143 (44)	41 (35)	16 (28)	
Occupation					0.141
Full-time	164 (33)	112 (34)	36 (30)	16 (28)	
Part-time	78 (16)	43 (13)	26 (22)	9 (15)	
Self-employed	37 (7)	28 (9)	6 (5)	3 (5)	
Housewife	141 (28)	89 (27)	33 (28)	19 (33)	
Retired	20 (4)	18 (6)	1 (1)	1 (2)	
Unemployed	57 (11)	32 (10)	16 (14)	9 (15)	
Other	3 (1)	2 (1)	0 (0)	1 (2)	
Region					
Hokkaido	26 (5)	15 (5)	4 (3)	7 (12)	0.038
Tohoku	23 (5)	17 (5)	4 (3)	2 (3)	0.645
Kanto	230 (46)	147 (45)	53 (46)	30 (52)	0.646
Chubu	63 (13)	45 (14)	10 (8)	8 (14)	0.303
Kansai	95 (19)	58 (18)	30 (25)	7 (12)	0.073
Chugoku	21 (4)	14 (4)	7 (6)	0 (0)	0.180
Shikoku	11 (2)	6 (2)	2 (2)	3 (5)	0.259
Kyushu	31 (6)	22 (7)	8 (7)	1 (2)	0.323
Annual individual income					
< 3.7 M Yen	286 (57)	173 (53)	73 (62)	40 (69)	0.272
3.7–7.7 M Yen	105 (21)	74 (23)	22 (19)	9 (15)	
> 7.7 M Yen	45 (9)	34 (11)	7 (6)	4 (7)	
I don’t know	64 (13)	43 (13)	16 (13)	5 (9)	
Time since diagnosis (mean ± SD)	10.67 ± 8.63	10.45 ± 8.99	11.07 ± 8.07	11.12 ± 7.63	0.242
≤ 5 years	165 (33)	111 (34)	38 (32)	16 (28)	
6–10 years	146 (29)	101 (31)	27 (23)	18 (31)	
> 10 years	189 (38)	112 (35)	53 (45)	24 (41)	
Current Medication					
Painkillers (NSAIDs/oral pain medication)	80 (16)	39 (12)	27 (23)	14 (24)	0.005
Steroid	110 (22)	66 (20)	29 (25)	15 (26)	0.481
DMARDs					0.112
csDMARDs	329 (66)	214 (66)	79 (67)	36 (62)	
csDMARDs + biologic agent	113 (23)	65 (20)	30 (25)	18 (31)	
Biologic agent	58 (12)	45 (14)	9 (8)	4 (7)	
Functional impairment					
J-HAQ score (mean ± SD)	0.40 ± 0.81	0.26 ± 0.55	0.52 ± 0.78	0.98 ± 1.50	<0.001
Comorbidity with depression					
Depression	25 (5)	7 (2)	6 (5)	12 (21)	<0.001
Comorbidity other than depression					
Hypertension	79 (16)	52 (16)	16 (14)	11 (19)	0.638
High cholesterol	42 (8)	24 (7)	11 (9)	7 (12)	0.458
Diabetes	26 (5)	15 (5)	10 (9)	1 (2)	0.122
Migraines	11 (2)	0 (0)	5 (4)	6 (10)	<0.001
Heart condition	8 (2)	3 (1)	2 (2)	3 (5)	0.060
Anxiety	8 (2)	3 (1)	2 (2)	3 (5)	0.060

*N* number, *%* percentage, *SD* standard deviation, *M* Million, *NSAID* nonsteroidal anti-inflammatory drugs, *RA* rheumatoid arthritis, *DMARDs* disease modifying anti-rheumatic drugs, *csDMARDs* conventional synthetic disease modifying anti-rheumatic drugs, *J-HAQ* Japanese version of the Stanford Health Assessment Questionnaire

### Depression prevalence

Table 1 reveals the prevalence of depression among the 500 People with RA analyzed across a number of demographic characteristics. Overall, 176 (35%) had depressive symptoms, whereas only 25 (5%) had been officially diagnosed with depression or visited physicians due to depression, suggesting depression might be under-diagnosed or under-reported in people with RA.

The average age of patients with no depression based on self-report PHQ-9 assessment was 56.04 years. By comparison, the average age of patients with mild depression was 51.57 years, and that of patients with moderate to severe depression was 49.93 years, suggesting that younger people with RA are more susceptible to depression. Among people with RA, the majority of major depression patients were female and married. Comorbidity conditions, except for migraine and heart conditions (for example chronic heart failure and ischemic heart disease), were not different between patients with depression and those without.

Painkillers were taken more in patients with depression than in those without (23% vs 12%, *P* = 0.005). No difference was found in treatment of steroid, csDMARDs and biologic agents.

Among the 324 patients who showed no signs of depression, the average J-HAQ score was 0.26, whereas the score among those with mild depression was 0.52 and those with moderate to severe depression had an average J-HAQ score of 0.98 (*P* < 0.001), indicating that the greater physical disability patients experience greater depression.

### Determinant of depression among people with RA

Table 2 shows the results of multivariable regression analysis for the determinants of depressive conditions among Japanese people with RA. A negative correlation with the prevalence of depression was found for age, meaning younger patients were more likely to experience depression, with a corresponding odds ratio (ORs) of 0.96 [95% confidence interval (CI); 0.94–0.98]. Higher education was also negatively correlated with depression, meaning more education resulted in less depression (ORs, 0.61 (CI 0.38–1.00) for a bachelor’s degree or higher. Another negative correlation was related to the biologic agent monotherapy (ORs, 0.36 (0.17–0.75)). People with RA with high J-HAQ score also had a high probability of developing depression (ORs 1.86: CI 1.40–2.48)). In summary, people with RA more likely to experience depression are those who are younger, less educated, have greater functional impairment, and who are treated with csDMARDs alone.

**Table 2:** Determinants of depression among RA patients

Characteristics	Univariate analysis		Multivariate analysis	
	ORs (95%CI)	*P*-value	ORs (95%CI)	*P*-value
Age	**0.95 (0.93–0.97)**	**<0.001**	**0.96 (0.94–0.98)**	**0.001**
Gender (Reference: Male)				
Female	**1.59 (1.07–2.38)**	**0.023**	0.86 (0.49–1.51)	0.599
Marital status (Reference: Single)				
Married	**0.52 (0.34–0.80)**	**0.003**	0.68 (0.40–1.17)	0.168
Highest Education (Reference: High school or less)				
College	0.91 (0.51–1.47)	0.690	0.89 (0.53–1.47)	0.639
Bachelor’s degree or higher	**0.57 (0.38–0.87)**	**0.010**	**0.61 (0.38–1.00)**	**0.050**
Occupation (Reference: Full-time)				
Part-time	**1.63 (0.96–2.78)**	**0.072**	1.26 (0.68–2.35)	0.459
Self-employed	0.70 (0.31–1.58)	0.396	0.85 (0.35–2.06)	0.725
Housewife	1.28 (0.81–2.05)	0.289	1.09 (0.59–2.00)	0.781
Retired	**0.25 (0.06–1.10)**	**0.067**	0.46 (0.94–2.30)	0.348
Unemployed	**1.69 (0.93–3.08)**	**0.086**	1.14 (0.55–2.37)	0.728
Other	1.60 (0.12–19.85)	0.715	1.48 (0.08–25.86)	0.787
Region				
Hokkaido	1.71 (0.77–3.21)	0.212		
Tohoku	0.64 (0.25–1.65)	0.361		
Kanto	1.10 (0.77–1.59)	0.584		
Chubu	0.75 (0.42–1.34)	0.333		
Kansai	1.09 (0.70–1.71)	0.692		
Chugoku	0.79 (0.32–1.92)	0.600		
Shikoku	1.88 (0.57–6.19)	0.296		
Kyushu	0.68 (0.31–1.48)	0.336		
Annual individual income (Reference <3.7 M Yen)				
3.7–7.7 M Yen	**0.63 (0.39–1.01)**	**0.058**	0.78 (0.46–1.34)	0.058
> 7.7 M Yen	**0.50 (0.24–1.03)**	**0.059**	0.75 (0.31–1.79)	0.059
Time since diagnosis (Reference: ≤5 years)				
6–10 years	0.96 (0.60–1.54)	0.872	0.99 (0.59–1.65)	0.964
> 10 years	**1.40 (0.91–2.13)**	**0.124**	1.13 (0.69–1.87)	0.624
J-HAQ score	**2.01 (1.59–2.53)**	**<0.001**	**1.86 (1.40–2.48)**	**<0.001**
Comorbidity				
Hypertension	0.99 (0.60–1.64)	0.986		
High cholesterol	1.46 (0.78–2.72)	0.233		
Diabetes	1.15 (0.54–2.45)	0.716		
Migraines	**13.23 (4.29–40.81)**	**<0.001**	9.27 (0.99–32.19)	0.087
Heart condition	**3.84 (1.01–14.61)**	**0.048**	4.18 (0.95–18.30)	0.058
Anxiety	**3.84 (1.01–14.61)**	**0.048**	2.20 (0.47–10.27)	0.317
Current medication				
Pain killer (NSAIDs/oral pain medication)	**2.11 (1.34–3.34)**	**0.001**	1.60 (0.94–2.72)	0.084
Steroid	1.30 (0.85–1.98)	0.228		
DMARDs (Reference: csDMARDs)				
csDMARDs + biologic agent	**1.40 (0.92–2.15)**	**0.117**	0.82 (0.49–1.37)	0.459
Biologic agent	**0.54 (0.28–1.05)**	**0.067**	**0.36 (0.17–0.75)**	**0.007**

Bold numbers indicate significance. Univariate analysis: significant *p* value < 0.2, multivariate analysis (stepwise approach): significant value = 0.05. *ORs* odds ratio, *CI* confidence interval, *RA* rheumatoid arthritis, *M* Million, *NSAID* nonsteroidal anti-inflammatory drugs, *DMARDs* disease modifying anti-rheumatic drugs, *csDMARDs* conventional synthetic disease modifying anti-rheumatic drugs, *J-HAQ* Japanese version of the Stanford Health Assessment Questionnaire

## Discussion

The results of this study reveal that one third of Japanese people with RA might potentially have depression as assessed by PHQ-9 while only 5% are officially diagnosed. Additionally, younger, less educated, more functionally impaired patients, and patients who are being treated with csDMARDs alone are more likely to have depressive syptoms. These findings are important to increase awareness of rheumatologist regarding depression. Furthermore, the results call for multidisciplinary treatment teams that do not only focus on the treatment of physical symptoms but also take into account a patient’s psychological condition. This approach will bring together the skills and knowledge of all team members which may may comprise case managers, pharmacists, physical and occupational therapists, social workers, physiatrists, orthopedists, or other health professionals to assess and manage care for the individual patient’s needs [22].

The key finding of this study is that a potential significant under-reporting of depression among Japanese people with RA might exist. The prevalence of depression among people with RA has been reported in previous studies worldwide [5], ranging from 15% up to 39%, and our finding of 35% by PHQ-9 is consistent with those results. Severe depressive symptom (PHQ ≥ 10) was less frequent in this study (11%) than the frequency reported in Asians and Pacific Islanders reported in 2009 (36%), which might be due to the development of treatment along with less physical functional impairment. However, there was still discrepancy in the prevalence between PHQ-9 and the official diagnosis. One explanation of the under-reporting of depression might be related to Asian cultural factors. People in Asian societies such as China and Japan are less likely to speak openly about depression due to the stigma attached to it as well as the need to maintain perceived strength of character [23]. However, differences in prevalence can be found when using different measurement instruments. For example, the point prevalence of major depressive disorders in Japanese people with RA was 6.8% when using the Mini-International Neuropsychiatric Interview [24]. This observation makes it the more important for physicians and all healthcare professional who are members of multidisciplinary team in Asia to be highly attuned to potential depression when treating their people with RA.

In this study, people with comorbid RA and depression tended to be younger than people without depression. This comparison is evident as well in the national Japanese survey conducted by the Ministry of Health, Labor, and Welfare in 2014 [25] and an Japanese employee survey conducted by the Northern-Japan Occupational Health Promotion Centers Collaboration Study for Mental Health [26]. Additionally, the results of the present study showed a positive association between depression and migraine and heart condition comorbidities. Several studies reported the relationship of depression with cardiovascular disease [27, 28], which is well known to be correlated with RA [29, 30].

Several studies show a positive correlation between depression and RA disease activity scores such as disease activity score for 28-joint (DAS28) or clinical disease activity index (CDAI) [31, 32]. Although the current study did not measure disease severity by such composite measures due to the limitations of online survey, people with RA were asked to rate their functional disability using J-HAQ [18], which closely correlates to both DAS28 and CDAI [33]. The results indicate that a higher J-HAQ score was correlated with depression. J-HAQ can measure a variety of functional limitations caused not only by inflammatory disease activity but also by joint damage and long-term disability. The resulting loss of valued activities has been shown to be a strong predictor of depression in patients with RA [13, 34]. Additionally, systemic inflammation that also causes functional disability is associated with, causes, or contributes to depressive symptoms experienced during the course of disorders that include chronic inflammation [35]. Patients with major depression have increased Interleukin 6 (IL-6) [36]. concentrations and pro-inflammatory tumor necrosis factor-alpha (TNF-α) [37] in serum, plasma, or both. A recent meta-analysis showed raised inflammatory markers such as IL-6 or C-reactive protein (CRP) are significantly associated with the subsequent development of depressive symptoms, which supports the hypothesis that there is an association between the inflammation and depression [38]. Consequently, medications that result in lower IL-6 and TNF-α level might have a direct positive impact on treating depression. Our findings also showed that patients receiving biologic agents had lower probability of developing depression compared to patients treated with csDMARDs alone, which can also be explained by the mechanism of the reduction of cytokine level that might be linked to depression [38–40]. These findings suggest that further research on the connection between biologic treatment and depression is needed.

There were several limitations to the present study. First, the analysis was based on a cross-sectional survey. As this was a single sample, the study was unable to demonstrate a causal relationship or account for changes in perception that might occur over time. Second, the study did not show that the documented depression condition was the direct consequence of the patient’s RA condition – other factors in patients’ lives could be the cause. Third, the exact disease activity was not completely known because this was an online survey consisting of patients reports. This hampered the analysis for the direct relationship between disease activity and depression. Furthermore, this was an online survey which might not be representative of the overall Japanese RA population. Usually, people that are more familiar with the Internet take part in online surveys which are in turn younger and probably better educated. On the other hand, the average age of our sample (54.28 years) does not differ much from a recent Japanese claims database analysis with more than 16,000 RA patients [41]. In that study the average patient age was 53.96 years which makes us believe that the potential selection bias in our sample is low. Last, there is a possibility of diagnostic overshadowing if some physical symptoms of RA are misattributed to depression. An example is feeling tired or having little energy which are both symptoms of depression as well as RA. This can potentially lead to an overestimation of depression in RA.

## Conclusions

In conclusion, the results of this study suggest that depression among Japanese people with RA is potentially under-reported and under-diagnosed. Rheumatologists should take particular care in assessing the psychological status of people with RA, particularly those susceptible to depression –younger patients, patients with greater functional impairment, and patients with a treatment regimen of csDMARDs alone.

## Additional file

Additional file 1: Reviewer reports and AU response to reviewers. (DOCX 18 kb)

## Abbreviations

CDAI: Clinical disease activity index
CI: Confidence interval
CRP: C-reactive protein
csDMARDs: Conventional synthetic disease modifying anti-rheumatic drugs
DAS28: Disease activity score for 28-joint
DMARDs: Disease modifying anti-rheumatic drugs
IL-6: Interleukin 6
J-HAQ: Japanese version of the Stanford Health Assessment Questionnaire
ORs: Odds ratio
PHQ-9: The Patient Health Questionnaire with 9 items
RA: Rheumatoid arthritis
TNF-α: Tumor necrosis factor-alpha
