# Supplementary material for: The underrated prevalence of depression in Japanese patients with rheumatoid arthritis - evidence from a Nationwide survey in Japan
Source: BMC Rheumatol. 2017 Nov 28;1:5. doi: 10.1186/s41927-017-0003-6 (PMC6383594; doi:10.1186/s41927-017-0003-6)
Supplement: Supplementary file 1 — Reviewer reports and AU response to reviewers. (DOCX 18 kb) [file 41927_2017_3_MOESM1_ESM.docx]

**Open Peer Review summary**

Pre-publication versions of this article, reviewer reports and author comments to reviewers are available by contacting [info@biomedcentral.com](mailto:info@biomedcentral.com).

| Original submission |  |  |
| --- | --- | --- |
| 30 May 2017 | Submitted | Original manuscript |
| 9 July 2017 | Reviewed | Reviewer report – Mwidimi Ndosi |
| 10 July 2017 | Reviewed | Reviewer report – Mary Margaretten |
| 10 August 2017 | Author responded | Author comments – Jörg Mahlich |
| Resubmission – Version 2 |  |  |
| 10 August 2017 | Submitted | Manuscript version 2 |
| 29 August 2017 | Author responded | Author comments – Jörg Mahlich |
| Resubmission – Version 3 |  |  |
| 29 August 2017 | Submitted | Manuscript version 3 |
| 15 September 2071 | Author responded | Author comments – Jörg Mahlich |
| Resubmission – Version 4 |  |  |
| 15 September 2017 | Submitted | Manuscript version 4 |
| 20 September 2017 | Author responded | Author comments – Jörg Mahlich |
| Resubmission – Version 5 |  |  |
| 20 September 2017 | Submitted | Manuscript version 5 |
| Publishing |  |  |
| 19 October 2017 | Editorially accepted |  |
| 28 November 2017 | Article published | 10.1186/s41927-017-0003-6 |

### How does Open Peer Review work?

Open peer review is a system where authors know who the reviewers are, and the reviewers know who the authors are. If the manuscript is accepted, the named reviewer reports are published alongside the article. Pre-publication versions of the article are available by contacting [info@biomedcentral.com](mailto:%20mailto:info@biomedcentral.com).

You can find further information about the peer review system at <https://bmcrheumatol.biomedcentral.com/about#peer-review+policy>
